# Orofacial Pain during Mastication in People with Dementia: Reliability Testing of the Orofacial Pain Scale for Non-Verbal Individuals

**DOI:** 10.1155/2016/3123402

**Published:** 2016-02-08

**Authors:** Merlijn W. de Vries, Corine Visscher, Suzanne Delwel, Jenny T. van der Steen, Marjoleine J. C. Pieper, Erik J. A. Scherder, Wilco P. Achterberg, Frank Lobbezoo

**Affiliations:** ^1^Department of Oral Health Sciences, Academic Centre for Dentistry Amsterdam (ACTA), University of Amsterdam and VU University Amsterdam, Research Institute MOVE Amsterdam, Gustav Mahlerlaan 3004, 1081 LA Amsterdam, Netherlands; ^2^Department of Clinical Neuropsychology, VU University Amsterdam, Van der Boechorstraat 1, 1081 BT Amsterdam, Netherlands; ^3^Department of General Practice & Elderly Care Medicine, VU University Medical Center (VUmc), Van der Boechorststraat 7, 1081 BT Amsterdam, Netherlands; ^4^Department of Public Health and Primary Care (PHEG), Leiden University Medical Center (LUMC), Hippocratespad 21, 2300 RC Leiden, Netherlands

## Abstract

*Objectives*. The aim of this study was to establish the reliability of the “chewing” subscale of the OPS-NVI, a novel tool designed to estimate presence and severity of orofacial pain in nonverbal patients.* Methods*. The OPS-NVI consists of 16 items for observed behavior, classified into four categories and a subjective estimate of pain. Two observers used the OPS-NVI for 237 video clips of people with dementia in Dutch nursing homes during their meal to observe their behavior and to estimate the intensity of orofacial pain. Six weeks later, the same observers rated the video clips a second time.* Results*. Bottom and ceiling effects for some items were found. This resulted in exclusion of these items from the statistical analyses. The categories which included the remaining items (*n* = 6) showed reliability varying between fair-to-good and excellent (interobserver reliability, ICC: 0.40–0.47; intraobserver reliability, ICC: 0.40–0.92).* Conclusions*. The “chewing” subscale of the OPS-NVI showed a fair-to-good to excellent interobserver and intraobserver reliability in this dementia population. This study contributes to the validation process of the OPS-NVI as a whole and stresses the need for further assessment of the reliability of the OPS-NVI with subjects that might already show signs of orofacial pain.

## 1. Introduction

Statistics Netherlands predicts that the percentage of elderly people (i.e., 60 years of age or older) will rise from 15% at present to 25% by 2040 [1]. This is not just a national Dutch phenomenon. Globally, the United Nations predict proportions of elderly rising to approximately 20%, thus doubling the percentage of people over 60, and even more so in more developed regions where life expectancy is higher (up to 40% regionally) [2].

One of the major challenges with an ageing population is dementia, of which a prevalence up to 7% in people over 60 is reported [3]. Many of these individuals' functions deteriorate to such a level that self-care is no longer possible. Although in some cases the care for people with dementia is supported by their families, others eventually come to live in a nursing home. Many people's functions continue to deteriorate until verbal communication is no longer possible [4]. The progressive decline in communicative abilities may hamper pain assessment in people with dementia, especially when it comes to orofacial pain [5]. In 2011, Lobbezoo et al. [5] emphasized that the existing diagnostic tools for establishing the intensity of pain in nonverbal elderly people with dementia are not appropriate for the assessment of dental or orofacial pain. In the same article, it was noted that there is a lack of research dealing with the assessment of orofacial pain in nonverbal people with dementia. The same is true for the literature on management of orofacial pain in this group [5, 6].

During the international and interdisciplinary process towards the development the Pain Assessment in Impaired Cognition metatool [4], the importance of developing a specific orofacial pain assessment tool was noted. Unfortunately, even though the recently developed Orofacial Mobilization-Observation-Behavior-Intensity-Dementia Pain Scale was proposed as a tool to assess the intensity of orofacial pain in individuals with dementia, it proved to be unreliable [7]. This led to the development of the “Orofacial Pain Scale for Non-Verbal Individuals” (OPS-NVI) [4, 8, 9]. This instrument is meant to assess the presence of possibly pain-related nonverbal communication, such as facial expressions, body movements, and vocal expressions. The patient's behavior is monitored during four types of activities, namely, “resting,” “drinking,” “chewing,” and “oral care,” and the intensity of the possible orofacial pain is scored as well. For the OPS-NVI, a reliability and validity assessment has yet to be performed. The present study therefore focuses on testing the reliability of the “chewing” subscale of the OPS-NVI by the assessment of video recordings of older people with dementia during their meal.

## 2. Material and Methods

### 2.1. Study Sample

For this study, video clips were used. These clips were part of the data set recorded in relation to the STA-OP!-protocol [10]. The video clips were recorded at various nursing homes throughout the Netherlands and consisted of audiovisual material of residents of these homes recorded during mealtime. Participating nursing homes met the following criteria:

- Management was willing to give permission for at least one psychogeriatric unit to participate.
- No major organizational changes or building activities were planned or performed in the study period.

For the residents to be preselected for enrollment, the inclusion criteria were

- presence of moderate to severe cognitive impairment according to the Global Deterioration Scale (GDS), that is, a score of 5, 6, or 7 [11],
- absence of chronic psychiatric diagnoses other than a dementia-associated diagnosis.

Both criteria were assessed by elderly care physicians who are part of the staff of Dutch nursing homes.

Informed proxy consent for the videotaping and use of the videotapes in the STA-OP!-study and related studies was obtained from family and/or caregivers for every included resident. The Medical Ethics Review Committee of the VU University Medical Center Amsterdam approved the protocol (registration number 2009/119).

After preselection, in order to be enrolled into the study, an additional inclusion criterion was the presence of “clinically significant symptoms of pain” and/or “difficult behavior,” defined as

- Cohen-Mansfield Agitation Inventory (CMAI) [12] score ≥44;
- Neuropsychiatric Inventory-Nursing Home Version (NPI-NH) [13] score ≥4 on every respective item; or
- indication of clinically relevant pain according to the Minimum Data Set of the Resident Assessment Instrument pain scale (MDS-RAI) (MDS-RAI pain scale ≥2) [14].

The degree of cognitive deterioration was measured according to the MDS-Cognitive Performance Scale (CPS) [15]. The CPS is a seven-category index, ranging from cognitively intact to very severely impaired. The index is categorized by combining the three severe categories as “severe” cognitive deterioration, the middle two categories as “moderate” deterioration, and the remaining two categories as “normal” cognitive performance or only mild deterioration. The CPS scale has shown excellent agreement with the Mini-Mental State Examination (MMSE) in the identification of cognitive impairment in research [16]. The CPS score's mean and standard deviation are shown in Table 1.

Comorbidity was assessed with the MDS-RAI comorbidity list, which contains the following groups of diseases: endocrine diseases, visual impairments, cardiovascular diseases, psychiatric disorders, pulmonary diseases, diseases of musculoskeletal system, neurological diseases (without Alzheimer disease or other types of dementia), infection in the last 7 days, and other [14]. Information on comorbidity is included in Table 1.

### 2.2. Procedure

The OPS-NVI consists of four subscales wherein different activities are assessed, namely, “resting” (I), “drinking” (II), “chewing” (III), and “oral care” (IV). Each subscale contains a total of 16 items of observed behavior that are classified into four categories, namely, “facial activities” (1), “body movements” (2), “vocalizations” (3), and “specific behavior”(4). All categories and items therein are identical for each subscale. For this study, only the “chewing” subscale was used. The items of observed behavior are shown in the following.

*Items for Observed Behavior of the “Chewing” Subscale of the OPS-NVI*

*(1) Facial Activities*

(Q1) Frowning: lowering and drawing brows together.
(Q2) Narrowing or closing eyes: narrowed eyes with tension around the eyes, not just blinking.
(Q3) Raising upper lip: upper lip raised, nose may be wrinkled.
(Q4) Opened mouth: the lips are parted and jaw is dropped.
(Q5) Tightened lips: lips are pressed together and appear more narrow.

*(2) Body Movements*

(Q6) Resisting care: resisting care, being uncooperative.
(Q7) Guarding: protecting affected area, holding body part, avoiding touch, and moving away.
(Q8) Rubbing: tugging or massaging affected area.
(Q9) Restlessness: fidgeting, wringing hands, and rocking back and forth.

*(3) Vocalizations*

(Q10) Using offensive words: cursing, sweating, or using foul language.
(Q11) Using pain-related words: using pain words, like “ouch,” “ow,” or “that hurts.”
(Q12) Screaming/shouting: using a loud voice to express sounds/words.
(Q13) Groaning: making deep, inarticulate sounds.

*(4) Specific Behavior*

(Q14) Restricting jaw movement: making smaller jaw movements than possible.
(Q15) Refusing prosthetics: removing prosthetics again and again.
(Q16) Drooling: flowing of saliva outside the mouth.

To complete the OPS-NVI for the purpose of this study, an adaptation of the standard instructions of the OPS-NVI was given to the observers:

1. Observe the behavior of the client while chewing:
  - Observe the activity for 3 minutes* or* for the length of the activity. Segments where no activity is shown can be skipped.
2. For each item, tick off the appropriate box:
  - Y = Yes, I saw this behavior.
  - N = No, I did not see this behavior.
  - N/A = Not Applicable; it was not possible to score this behavior, because the client was not able to perform this behavior (not: not visible. In that case, tick off “No”).
3. Rate the* estimated* pain intensity with a number between 0 and 10:
  - 0 is no pain and 10 is pain as bad as it possibly could be.
  - Rate what* you think* is the experienced pain intensity.

For this study, a total of 321 video clips were collected. Of these, 84 were not used. This was because in 83 cases, no or hardly any masticatory movement was detected, while one clip was removed from the data set because, in retrospect, the person had a possible alcohol-related dementia diagnosis, which did not meet the inclusion criteria as described in the STA-OP!-protocol. This yielded a total of 237 video clips to be observed, with a total of 153 subjects. From these, 69 subjects featured in only one video clip, whereas 84 featured in two clips. The subjects that were filmed twice were recorded with a 3-month interval (12-13 weeks) in between both recordings [10]. There were 109 women and 44 men, with a mean age of 83.3 (SD: 7.1; range: 63.8–102.4), as shown in Table 1.

The video clips featured residents during their mealtime. The clips were recorded with audiovisual recording equipment (JVC brand, type Everio G Series nr. GZ-MG575, Yokohama, Japan). The camera was placed in such a way that the resident's face was shown, nearly all masticatory movements were clearly visible, and vocalizations were clearly heard over the course of the clip. If the resident moved during the recording, the camera position was adjusted accordingly. The duration of the clips varied between 3 and 5 minutes.

### 2.3. Reliability Assessment

Two observers, both sixth year dental students at the Academic Centre for Dentistry Amsterdam (ACTA), were given a training by an experienced user of the OPS-NVI and were instructed to individually observe the behavior of the participants and estimate the pain intensity with the OPS-NVI for every clip (*t*0), followed by a period of 6 weeks of no observation. After this period, the observers were instructed to complete the OPS-NVI again for every clip (*t*1).

### 2.4. Statistical Analysis

To establish the reliability of the “chewing” subscale of the OPS-NVI, the interobserver and intraobserver reliability were assessed by analyzing the test-retest reliability for individual items of the instrument. The sum scores of the items per category and the interobserver and intraobserver reliability of the estimated pain score were also analyzed. For all interobserver reliability analyses, the *t*0-measurements of both observers were used.

In cases where the database showed a bottom or ceiling effect for an item, meaning that the item was scored in less than 5% or more than 95% of the cases, it was decided that the item was excluded from the statistical analyses. Thus, items with a Yes or No count <12 were excluded.

The interobserver and intraobserver reliability of the item scores were analyzed using Intraclass Correlation Coefficients (ICCs). The interobserver and intraobserver reliability of the sum scores of the included items per category and of the estimated pain scores were also estimated by ICC. ICCs < 0.4 were considered poor, ICCs between 0.4 and 0.75 fair-to-good, and ICCs > 0.75 excellent [17]. The confidence interval was calculated with a 95% confidence level. The percentage agreement for the item scores was also determined.

Probability levels of *p* < 0.05 were defined as statistically significant. All statistical analyses were performed using the SPSS software package version 20.0 (IBM, Armonk, NY, USA, 2011).

## 3. Results

As shown in Table 2, a total of ten items were excluded from the statistical analyses, because there was hardly any variability in observed behavior. As a result, the category “vocalizations” was not used in the further analyses. For most cases, excluded items were scored “No,” with the exception of (Q4), which was excluded from the analyses because in all cases subjects opened their mouths as part of their chewing activities.

Table 3 shows the intraobserver and interobserver reliability and percentage agreement per included item. The table clearly shows a discrepancy between the different observations: the intraobserver reliability of observer 1 ranges from fair-to-good to excellent, while the intraobserver reliability and interobserver reliability of observer 2 range from poor to fair-to-good.

In Table 4, where intraobserver and interobserver reliability per category as well as pain intensity estimations are shown, a similar discrepancy between the two intraobserver reliabilities is noted. However, the reliability per category seems to be slightly higher than the reliability per item.

## 4. Discussion

The aim of this study was to assess interobserver and intraobserver reliability of the “chewing” subscale of the OPS-NVI, with patient and environment standardized through video recordings. When analyzing the video clips, the two observers reported clear bottom and ceiling effects, meaning that there were a considerable number of cases in which an item was observed in less than 5% or more than 95% of the cases. This might be due to the fact that although there was preselection for “clinically significant symptoms of pain” and/or “difficult behavior,” as defined by the STA-OP!-protocol inclusion criteria [10], there was no specific selection of cases with probable orofacial pain. Therefore, it was decided that all items that were considered noncontributing to orofacial pain for this population were excluded. While the category “facial activities” only lost a single item (namely, “opened mouth,” which was excluded because this behavior is always present while eating), the category “vocalizations” was completely excluded, and of the categories “body movements” and “specific behavior” only one item was maintained. These results suggest that the “chewing” subscale of the OPS-NVI might be reduced to the remaining 6 items, which would facilitate its use in daily practice.

The discrepancies in the intraobserver reliability between the two observers, as shown in Tables 3 and 4, could be explained as follows. The instructions to the observers were to first score the presence of different items of behavior, regardless of whether the observer thought it was related to orofacial pain. Looking at, for example, the first category, namely, “facial activities” ((Q1) to (Q5)), it indicates behavior that can also be present during masticatory movement without pain. This complicates the observations considerably. Within this context, when observing this behavior, the different observers apparently showed a different sensitivity for the more subtle facial movements, which show that the scoring of the OPS-NVI items is based on a subjective interpretation of observed behavior. To improve the reliability, a different set of instructions, for example, pictures of frowning and nonfrowning individuals that guide the decision to (not) score this specific item, could be developed.

From 84 people, two video fragments were available, because the clips were recorded as part of the STA-OP!-protocol and were therefore obtained at baseline and after 3 months [10]. It could be argued that this could have created observer bias within this study; that is, the pain score could have been based on recollection of previous film clips featuring the same person rather than on independent observations. This could have led to an overestimation of reliability. However, the time between both recordings was relatively long. Taking this into account, along with the fluctuating nature of most painful conditions, it was therefore decided that even though a person featured twice in the database, both clips could be considered as independent of each other.

### 4.1. Strengths and Limitations

A strength of the present study is the large number of video clips (*n* = 237) included in the sample, which contributed greatly to the power of the study. Furthermore, not only did using video clips provide an efficient way to collect a lot of data in a short period of time, but also it offered the possibility of assessing the intraobserver reliability, which would otherwise have been impossible.

A limitation might be that observing video clips is not the same as real-life observation. Although most clips were at most 5 minutes long, it is still a limited period of time. This may have resulted in the observed bottom and ceiling effects. Life observation over a longer period, that is, during the course of the entire meal, may have yielded a more accurate estimate of the presence of orofacial pain during mastication. However, longer observations create the risk of making the OPS-NVI more impractical to use and more difficult to implement on a large scale. This study could also have benefited from additional observers, since clear discrepancies between the ICC scores of observer 1 and observer 2, the former ranging from fair-to-good to excellent and the latter from poor to fair-to-good. The lack of a control group with subjects matched for age but without cognitive decline is also a potential limitation: in this study, establishing the presence and intensity of orofacial pain in the included subject is confounded not only by the use of a novel tool, but also by the fact that the subject suffers from severe cognitive decline. By including a control group, the latter will no longer be a confounding factor. It is therefore suggested that future studies into the reliability and validity of the OPS-NVI include a control group.

### 4.2. Implications

This study was performed to contribute to the reliability assessment of the “chewing” part of the OPS-NVI and also to its development as a whole. In the process of assessing the interobserver and intraobserver reliability, it was found that a total of ten items could be excluded from this subscale of the OPS-NVI, which makes it more concise and easier to use. Additional reliability assessments are required for the other subscales of the OPS-NVI (namely, resting, drinking, and oral hygiene). Following this, validity of the tool will also need to be assessed.

### 4.3. Conclusion

The Orofacial Pain Scale for Non-Verbal Individuals (OPS-NVI) is developed to improve the recognition of the presence and intensity of orofacial pain. In this study, it was used to assess pain in older people with dementia during their meal, for which the “chewing” subscale of the OPS-NVI was used. The categories within the “chewing” subscale of the OPS-NVI have a fair-to-good to excellent interobserver and intraobserver reliability. The outcomes stress the need for further assessment of the reliability of the OPS-NVI in subjects with more severe orofacial pain.

## Figures and Tables

**Table 1:** Descriptive and demographic data of subjects (*N* = 153).

	Mean	SD	*N*	Percentage
Age	83.3	7.1		
Male	81.1	7.6		
Female	84.1	6.7		
CPS score	4.4	1.3		
Comorbidity				
Endocrine^a^			46	30.1
Vision impairment^b^			25	16.3
Heart/cardiovascular disease^c^			86	56.2
Psychiatric/mood^d^			22	14.4
Lung disease^e^			22	14.4
Diseases of musculoskeletal system^f^			42	27.5
Neurological diseases^g^			38	24.8
Infection in the last 7 days^h^			6	3.9
Other^i^			21	13.7

SD: standard deviation.

CPS: Cognitive Performance Scale.

a = diabetes mellitus, hypothyroidism, and/or hyperthyroidism.

b = cataract, diabetic retinopathy, glaucoma, and/or macular degeneration.

c = arteriosclerotic disease, heart rhythm disorders, heart failure, hypertension, hypotension, peripheral vascular disease, and other.

d = anxiety disorder, depression, manic depression, and schizophrenia.

e = asthma, emphysema/COPD.

f = rheumatic diseases, hip fracture, amputation, osteoporosis, and pathologic bone fracture.

g = aphasia, cerebral palsy, stroke, hemiplegia/hemiparesis, paraplegia, multiple sclerosis, Parkinson disease, seizures, transient ischemic attack, traumatic brain injury, and quadriplegia.

h = pneumonia, respiratory tract infection, and urinary tract infection.

i = allergies, anemia, cancer, and renal failure.

**Table 2:** Proportion of positive observations of the OPS-NVI items by observer and assessment (*N* = 237 video clips).

	Observer 1		Observer 1		Observer 2		Observer 2	
	Assessment 1		Assessment 2		Assessment 1		Assessment 2	
	Count	Percentage	Count	Percentage	Count	Percentage	Count	Percentage
	Yes	%	Yes	%	Yes	%	Yes	%
(1) Facial activities								
(Q1) Frowning	135	57.0	137	57.8	143	60.3	135	57.0
(Q2) Narrowing or closing eyes	118	49.8	108	45.6	153	64.6	167	70.5
(Q3) Raising upper lip	65	27.4	54	22.8	124	52.3	117	49.4
(Q4) Opened mouth^*∗*^	237	100.0	237	100.0	237	100.0	237	100.0
(Q5) Tightened lips	143	60.3	138	58.2	175	73.8	189	79.8
(2) Body movements								
(Q6) Resisting care^*∗*^	1	0.4	1	0.4	1	0.4	0	0.0
(Q7) Guarding^*∗*^	0	0.0	0	0.0	3	1.3	2	0.8
(Q8) Rubbing^*∗*^	0	0.0	0	0.0	6	2.5	6	2.5
(Q9) Restlessness	7	2.9	6	2.5	12	5.1	4	1.6
(3) Vocalizations								
(Q10) Using offensive words^*∗*^	0	0.0	0	0.0	1	0.4	1	0.4
(Q11) Using pain-related words^*∗*^	0	0.0	0	0.0	0	0.0	1	0.4
(Q12) Screaming/shouting^*∗*^	2	0.8	3	1.3	2	0.8	2	0.8
(Q13) Groaning^*∗*^	2	0.8	2	0.8	2	0.8	5	2.1
(4) Specific behavior								
(Q14) Restricting jaw movement	36	15.2	41	17.3	29	12.2	34	14.3
(Q15) Refusing prosthetics^*∗*^	0	0.0	0	0.0	1	0.4	0	0.0
(Q16) Drooling^*∗*^	1	0.4	1	0.4	0	0.0	0	0.0

^*∗*^Excluded from further data analysis.

**Table 3:** Intraobserver and interobserver reliability per item.

Item	Intraobserver Observer 1			Intraobserver Observer 2			Interobserver Over assessments 1		
	ICC	Agreement (in %)	95% CI	ICC	Agreement (in %)	95% CI	ICC	Agreement (in %)	95% CI
(Q1) Frowning	0.64	82.3	0.56–0.71	0.48	74.7	0.38–0.57	0.50	75.5	0.40–0.59
(Q2) Narrowing or closing eyes	0.68	84.0	0.61–0.74	0.31	69.6	0.19–0.42	0.25	62.4	0.13–0.37
(Q3) Raising upper lip	0.61	85.2	0.52–0.68	0.35	67.5	0.24–0.49	0.30	64.1	0.15–0.43
(Q5) Tightened lips	0.80	90.3	0.75–0.84	0.29	74.7	0.17–0.40	0.35	70.5	0.23–0.46
(Q9) Restlessness	0.92	99.6	0.90–0.94	0.49	96.6	0.38–0.58	0.40	95.4	0.29–0.50
(Q14) Restricting jaw movement	0.77	93.7	0.71–0.81	0.40	86.1	0.29–0.50	0.41	86.1	0.30–0.51

ICC = Intraclass Correlation Coefficient.

CI = confidence interval.

**Table 4:** Intraobserver and interobserver reliability for the category sum scores as well as for the estimated pain intensity scale.

Category	Intraobserver Observer 1		Intraobserver Observer 2		Interobserver Over assessments 1	
	ICC	95% CI	ICC	95% CI	ICC	95% CI
Facial activities	0.76	0.70–0.81	0.49	0.38–0.58	0.41	0.25–0.54
Body movements	0.92	0.90–0.94	0.49	0.38–0.58	0.40	0.29–0.50
Vocalizations^*∗*^	N/A	N/A	N/A	N/A	N/A	N/A
Specific behavior	0.77	0.71–0.82	0.40	0.29–0.50	0.41	0.30–0.51

Pain intensity	0.81	0.76–0.85	0.58	0.49–0.66	0.47	0.36–0.56

ICC = Intraclass Correlation Coefficient.

CI = confidence interval.

^*∗*^All items from the category “vocalizations” were excluded from statistical analysis.
